# Manual of Physiology

**Published:** 1849-11

**Authors:** 


### ARTICLE II.

*Manual of Physiology*—By WILLIAM SENHOUSE KIRKES, M.D., Assisted by JAMES PAGET, Lecturer on General Anatomy and Physiology at St. Bartholomew's Hospital. With one Hundred and Eighteen Illustrations on wood: Philadelphia: Lea and Blanchard. 1849: pp. 552. For sale by S. C. Griggs & Co.

This is one of the best elementary works on Physiology within the reach of the student of Medicine. It is concise and yet sufficiently full to present the important facts of the science in a plain and perspicuous style. It contains enough of General and Microscopic Anatomy to give the reader a fair knowledge of their bearing on the development and functions of the various tissues and organs; and the closing chapter on Generation and Development, presents the modern discoveries and doctrines concerning Ovology and successive development with clearness and accuracy. The whole work is suitably illustrated by well executed wood cuts. Like all other works on the general subject of Human Physiology, it contains some doctrines which admit of criticism. But to discuss these with fairness or profit to the reader would require more time and space than we have of either, at our command, for the present number of the Journal. It is nevertheless a clear, concise, and truly elementary work; and therefore better adapted for a text-book, than some of the more voluminous and expensive works which are recommended for that purpose.

